# Bee Pollen Improves Muscle Protein and Energy Metabolism in Malnourished Old Rats through Interfering with the Mtor Signaling Pathway and Mitochondrial Activity

**DOI:** 10.3390/nu6125500

**Published:** 2014-12-01

**Authors:** Jérôme Salles, Nicolas Cardinault, Véronique Patrac, Alexandre Berry, Christophe Giraudet, Marie-Laure Collin, Audrey Chanet, Camille Tagliaferri, Philippe Denis, Corinne Pouyet, Yves Boirie, Stéphane Walrand

**Affiliations:** 1 INRA, UMR1019, Unité de Nutrition Humaine, CRNH Auvergne, Clermont-Ferrand F-63000, France; E-Mails: Jerome.Salles@clermont.inra.fr (J.S.); veronique.patrac@clermont.inra.fr (V.P.); alexandre.berry@clermont.inra.fr (A.B.); christophe.giraudet@clermont.inra.fr (C.G.); marie-laure.collin@clermont.inra.fr (M.-L.C.); audrey.chanet@clermont.inra.fr (A.C.); camille.tagliaferri@clermont.inra.fr (C.T.); philippe.denis@clermont.inra.fr (P.D.); corinne.pouyet@clermont.inra.fr (C.P.); boirie@clermont.inra.fr (Y.B.); 2 Clermont Université, Université d’Auvergne, Unité de Nutrition Humaine, BP 10448, Clermont-Ferrand F-63000, France; 3 Pollenergie, La Grabère, St Hilaire de Lusignan F-47450, France; E-Mail: nicolas.cardinault@gmail.com; 4 CHU Clermont-Ferrand, Clinical Nutrition Department, Clermont-Ferrand F-63003, France

**Keywords:** fresh bee pollen, body composition, skeletal muscle, protein synthesis, mitochondria

## Abstract

Although the management of malnutrition is a priority in older people, this population shows a resistance to refeeding. Fresh bee pollen contains nutritional substances of interest for malnourished people. The aim was to evaluate the effect of fresh bee pollen supplementation on refeeding efficiency in old malnourished rats. Male 22-month-old Wistar rats were undernourished by reducing food intake for 12 weeks. The animals were then renourished for three weeks with the same diet supplemented with 0%, 5% or 10% of fresh monofloral bee pollen. Due to changes in both lean mass and fat mass, body weight decreased during malnutrition and increased after refeeding with no between-group differences (*p* < 0.0001). Rats refed with the fresh bee pollen-enriched diets showed a significant increase in muscle mass compared to restricted rats (*p* < 0.05). The malnutrition period reduced the muscle protein synthesis rate and mTOR/p70S6kinase/4eBP1 activation, and only the 10%-pollen diet was able to restore these parameters. Mitochondrial activity was depressed with food restriction and was only improved by refeeding with the fresh bee pollen-containing diets. In conclusion, refeeding diets that contain fresh monofloral bee pollen improve muscle mass and metabolism in old, undernourished rats.

## 1. Introduction

Bee pollen has been used for years as an exceptionally nutrient-rich health supplement [1]. It comes from the pollen that collects on the bodies of bees. Fresh bee pollens contain high levels of proteins, amino acids, lipids, minerals, carbohydrates, vitamins and other compounds, such as lactic acid bacteria, incorporated in bee saliva that is important for fermentation of the product [2]. Bee pollens are also rich in flavonoid and phenolic compounds. Their antioxidant effects are largely related to their free radical scavenging activity [2,3].

Bee pollen has been recommended for use in different physiopathological conditions and is widely used in Chinese medical clinical practice. The German Federal Board of Health has officially recognized bee pollen as a medicine [4]. Bee pollen is often claimed to cure certain health problems, and there is evidence that it can improve microcirculation and dyslipidemia and prevent and control coronary heart disease and myocardial infarction [5,6]. Bee pollen is cytotoxic against tumor cells [7] and regulates immune activity [8], and it is recommended to enhance athletic performance, reduce the side effects of chemotherapy and improve allergies and asthma [9].

Protein-energy malnutrition is common in elderly people, especially in aged hospitalized patients [10] in whom malnutrition is an important factor of morbidity and mortality. Malnutrition can surface and/or worsen intrinsic age-related changes, such as digestive tract alterations [11], protein metabolism [12] and immunological abnormalities [13,14,15]. In elderly women, lean body mass, skeletal muscle function and immune status fail to adapt to low-protein intake [16]. In addition, aged rats accommodated less efficiently to a long-term dietary restriction than adult rats, particularly in terms of protein metabolism [14]. In old rats, malnutrition induced a dramatic loss of body weight and affected nitrogen balance and tissue protein content, especially at the splanchnic and muscle level [17,18]. It is therefore clear that on a molecular level, age-related malnutrition is accompanied by impaired protein metabolism.

Moreover, aged populations show a slow decline in the ability to recover from a malnourished state, *i.e.*, correction of the malnourished state is more difficult in elderly people than younger subjects [19,20]. We previously reported that in rats, as in humans, the response to refeeding is affected by aging [14,17,21]. Accordingly, investigations need to be developed to study nutritional strategies to counteract malnutrition as soon as possible and, thus, avoid potential irreversible lesions in aged populations. For instance, in old malnourished rats, nutritional and immunologic variables can be improved by adding a very high amount of protein very early to the refeeding diet [14]. Elderly malnourished people may therefore require higher amounts of protein during refeeding, possibly due to increased protein utilization by splanchnic tissues. Furthermore, previous studies have revealed that realistic and safe therapeutic approaches based on nutrient supplementation, e.g., amino acids, are able to improve muscle protein metabolism in the frail state related to malnutrition in aging [22]. In this context, using bee pollen, which contains high concentrations, not just of amino acids, but also other key nutrients, could offer a valuable alternative approach.

Prompted by the fact that nutritional supplements may be required to act as a starter of renutrition in older subjects, this study was designed to evaluate the efficiency of refeeding diets supplemented with specific fresh bee pollen formula in old malnourished rats. We investigated whether malnutrition-induced alterations in body weight and composition and muscle protein and energy metabolism were reversible in old rats receiving renutrition via two fresh pollen-enriched diets, *i.e.*, 5% bee pollen and 10% bee pollen supplementation.

## 2. Material and Methods

### 2.1. Animals

All rats and experimental procedures were used in accordance with the Clermont-Ferrand University (France) Institutional Ethics Committee (C2EA-02), and we are authorized by the French Ministry of Agriculture and Forestry to perform animal experiments. Forty-five old male Wistar rats were purchased from the Janvier breeding center (Le Genest-St-Isle, France). All rats were the same age (22 months old at the end of the experiment), came from the same batch and were bred under the same conditions throughout their lives. The rats were housed in individual cages under a 12-h light/12-h dark cycle at 22 °C with free access to water. During acclimatization, all of the rats were fed a standard diet (AIN93M) *ad libitum* for 2 weeks. Daily spontaneous intakes were determined in Week 2; old rats consumed 24 g/day.

### 2.2. Experimental Procedures

The compositions of the experimental diets are given in Table 1. Food-restricted rats were allowed to consume 50% (12 g/day) of their spontaneous intakes as measured during the second week of the acclimatization period. During dietary restriction, the old rats were fed a standard diet for 12 weeks according to our established lab protocol [14]. At the end of the dietary restriction program, all animals were refed *ad libitum* for 3 weeks with either the standard diet or a diet supplemented with 5 g/100 g (5%) or 10 g/100 g (10%) specific fresh monofloral bee pollen formula provided by Pollenergie. This patented formula is constituted from 3 specific entomophile monofloral bee pollens in a particular proportion. The composition of this fresh bee pollen formula is given in Table 2. Food was given in conical porcelain cups to avoid spillage, and food intake did not differ between groups during the refeeding period [23]. All rats in every group ate their entire ration. Control rats were fed the standard diet *ad libitum* throughout the experiment.

Animals were weighed on a weekly basis. Body composition was measured at the beginning and end of the experimental period using the Echo MRI method (EchoMRI-100 instrument, Houston, TX, USA). At the end of the experiment and after a 16-h fasting period, the rats were anesthetized with isoflurane, and blood was collected by abdominal aorta puncture. Immediately after sacrifice, tissues were collected, weighed, frozen in liquid nitrogen and stored at −80 °C until analysis.

**Table 1:** Composition of the diets.

g/100 g	Standard Diet	5% Pollen Diet	10% Pollen Diet
Casein	14	14	14
Cornstarch	46	41	36
Sucrose	28	28	28
Soybean oil	4	4	4
Cellulose	4	4	4
Vitamins and minerals mix	4	4	4
Bee pollen	0	5	10

**Table 2:** Composition and antioxidant value of fresh bee pollen formula.

Fresh Bee Pollen Formula	
*Proteins (g/100 g)*	16.2
EAA (g/100 g)	4.8
BCAA (g/100 g)	2.3
SAA (g/100 g)	0.6
*Carbohydrates (g/100 g)*	57.7
Lipids (g/100 g)	7.3
Saturated FA (% of total FA)	43.1
Monounsaturated FA (% of total FA)	16.1
Polyunsaturated FA (% of total FA)	40.8
Fiber (g/100 g)	11.4
Vitamin B_1_ (mg/100 g)	0.65
Vitamin B_2_ (mg/100 g)	0.76
Vitamin PP(mg/100 g)	7.05
Vitamin B_5_ (mg/100 g)	0.77
Vitamin B_6_ (mg/100 g)	2.38
Vitamin B_9_ (mg/100 g)	0.81
Vitamin C (mg/100 g)	42.7
Vitamin E (mg/10 0g)	12.9
Calories (kcal/100 g)	315.8
*Antioxidant value*	
FRAP (mmol/kg eq ascorbic acid)	25.8
ORAC (mmol/100 g eq trolox)	5.6

EAA: essential amino acids; BCAA: branched chain amino acids; SAA: sulfur amino acids; FA: fatty acids; FRAP: ferric reducing ability of plasma; ORAC: oxygen radical absorbance capacity value.

### 2.3. Blood Biochemistry

The serum level of insulin, leptin, adiponectin, resistin, tumor necrosis factor-alpha (TNFα), soluble TNFα receptors-1 and -2 (sTNF-R1, sTNF-R2), interleukin-1β (IL1β), IL6, prealbumin, orosomucoid and α2-macroglobulin were measured using ELISA kits (Alpco, distributor Eurobio, Les Ulis, France; R & D Systems, Lille, France; Abnova, distributor VWR, Strasbourg, France), following the manufacturer’s instructions.

### 2.4. In Vivo Protein Synthesis Measurement

To study muscle protein synthesis, we measured the rate of incorporation of a stable isotope, *i.e.*, a labeled amino acid (l-(1-^13^C) valine, Eurisotop, France), into muscle proteins using the flooding dose method. After an overnight fast, rats were injected subcutaneously with a large dose of l-(1-^13^C) valine (50% mol excess, 300 µM/100 g) to flood the precursor pool of protein synthesis. Tracer incorporation time was 50 min in all three groups. Pieces of plantaris muscle were used to isolate mixed proteins as previously described [24]. After protein hydrolysis, AA were derivatized, and l-(1-^13^C) valine enrichment in hydrolyzed proteins was measured by gas chromatography-combustion-isotope ratio mass spectrometry (Gas System; Fisons Instruments, Middlewich, UK). l-(1-^13^C) valine enrichments in tissue fluid were assessed by gas chromatography-mass spectrometry (Hewlett-Packard 5971A; Hewlett-Packard Co., Palo Alto, CA, USA) and used as precursor pool enrichment to calculate absolute synthesis rates (ASR). Two rats of each group were not tracer infused, but used to determine natural isotopic abundance in mixed muscle proteins. Protein ASR was calculated as:

ASR = {[(Eit − Ei0) × 100]/(Eip × *t*)} × TPC

where Eit is enrichment as atom % excess of valine-derived ^13^C in proteins at time *t* (minus basal enrichment Ei0), Eip is the mean enrichment in the precursor pool (tissue fluid l-(1-^13^C) valine), *t* is incorporation time and TPC is total protein content in mg. ASR is expressed as mg/h.

### 2.5. Western Blot Analysis

Fifty milligrams of frozen plantaris strips were minced and homogenized in ice-cold buffer (50 mM HEPES pH 7.4, 150 mM NaCl, 10 mM EDTA, 10 mM NaPPi, 25 mM β-glycerophosphate, 100 mM NaF, 2 mM Na orthovanadate, 10% glycerol, 1% Triton X-100) containing 1% of protease inhibitor cocktail (Sigma-Aldrich, Saint-Quentin Fallavier, France). Homogenates were centrifuged at 13,000× *g* for 10 min at 4 °C. Fifty micrograms of denaturated proteins were separated by SDS-PAGE and transferred to a polyvinylidene membrane (Millipore, Molsheim, France). Immunoblots were blocked with TBS-Tween-20 0.1% containing 5% dry milk and then probed with primary antibodies: anti-phospho mTOR (Ser2448), anti-total mTOR, anti-phospho S6 kinase (S6k, Thr389), anti-total S6k, anti-phospho eukaryotic-initiation factor 4-binding protein 1 (4E-BP1, Ser65) and anti-total 4E-BP1. All antibodies were purchased from Cell Signaling Technology (distributor Ozyme, Saint-Quentin-en-Yvelines, France). After several washes with TBS plus 0.1% Tween-20, the immunoblots were incubated with a horseradish peroxidase-conjugated secondary antibody (DAKO, Trappes, France). Luminescent visualization of secondary antibodies was done using ECL Western Blotting Substrate (Pierce, Thermo Fisher Scientific, Courtaboeuf, France) and an MF-ChemiBIS 2.0 imaging system (F.S.V.T., Courbevoie, France). Band densities were quantified using MultiGauge 3.2 software (Fujifilm Corporation, distributor FSVT, Courbevoie, France). An internal control was used on each gel to normalize signal intensities between gels. This internal control was a pool of all tested samples. Band densities were normalized to the internal control. The ratio of phosphorylated protein to total protein was calculated and then was expressed as a percentage of the phosphorylation rate measured for the internal control.

### 2.6. Mitochondrial Enzyme Activities

Fifty milligrams of frozen plantaris muscle were homogenized in a 5% ice-cold buffer containing 0.25 M sucrose, 2 mM EDTA and 10 mM Tris-HCl (pH 7.4) using a Potter-Elvehjem homogenizer. The homogenate was centrifuged at low speed (600× *g*), and the supernatant was collected, put through 5 freeze-thaw cycles and mixed thoroughly before measuring enzyme activities. Activities of citrate synthase (CS), a key enzyme of the Krebs cycle, 3-hydroxy acyl CoA dehydrogenase, a key enzyme of β-oxidation, and of complexes II to IV of the respiratory chain were spectrophotometrically assayed in the supernatant fraction, as previously described [25,26]. All measures were performed in triplicate. Enzyme activities were expressed in nmol/min/mg proteins.

### 2.7. Statistical Analysis

Data are given as the means ± SEM. Statistical analyses were performed using Statview version 5.0 (SAS Institute, Cary, NC, USA). ANOVA was used to examine differences between groups. When a significant effect was detected, a *post hoc* Fisher test was applied to locate pairwise differences between groups. Results were considered significant at the 5% level. Means sharing the same superscript letter are not significantly different from each other.

## 3. Results

### 3.1. Body and Tissue Weights

As expected, total body weight decreased significantly under the 12-week restricted diet (−2.5 g/day; *p* < 0.05 *versus* initial body weight and the control group, Table 3). The body weight of the control rats remained stable throughout the experiment (not shown). After three weeks of refeeding, the animals displayed a similar weight gain regardless of the diet used (33% to 36%, *p* < 0.05 *versus* food-restricted old rats). Body weight changes during food restriction and refeeding were due to changes in both lean mass and fat mass (Table 3). There were no between-rat differences in the weight of the total hindlimb muscles and the soleus (Table 3). However, gastrocnemius mass was decreased in food-restricted old rats compared to controls (*p* < 0.05). Interestingly, refeeding was able to restore gastrocnemius weight and even more effectively when fresh bee pollen was present in the diet (*p* < 0.05, 5% and 10% pollen *versus* food-restricted old rats). Old rats tended to show a reduced plantaris weight after the diet restriction period, and the refeeding diets supplemented with fresh bee pollen had a beneficial effect on plantaris weight (Table 3, *p* < 0.05 *versus* the restricted group). In agreement with the data on body composition, there was a strong reduction in the weight of visceral and subcutaneous adipose tissues after the food restriction period. Compared to food-restricted old rats, subcutaneous fat pad weight was not significantly different in old rats refed with the standard or 5% pollen diet, but was significantly higher in old rats refed with the 10% pollen diet. Similarly, visceral fat pad mass increased 10-fold for 10% pollen refed old rats compared to restricted animals, regardless of diet type (*p* < 0.05). Liver, heart and kidney weights were reduced by dietary restriction, then restored by refeeding (*p* < 0.05, Table 3), but with no effect of the type of diet used to refeed the animals.

**Table 3:** Body weight, body composition and tissue weight after dietary restriction and refeeding with fresh bee pollen-containing diets in old rats.

	Controls	Restricted	Refed Standard	Refed 5% Pollen	Refed 10% Pollen
Body weight (g)	629 ± 5 ^a^	418 ± 13 ^b^	570 ± 19 ^a^	556 ± 20 ^a^	560 ± 13 ^a^
Fat mass (g)	151 ± 16 ^a^	13 ± 3 ^b^	84 ± 11 ^c^	78 ± 9 ^c^	87 ± 5 ^c^
Fat-free mass (g)	491 ± 16 ^a^	385 ± 4 ^b^	493 ± 11 ^a^	487 ± 14 ^a^	482 ± 12 ^a^
Hindlimb muscle mass (g)	11.5 ± 0.7	10.8 ± 0.7	11.8 ± 1.1	12.2 ± 0.4	12.7 ± 0.6
Plantaris (mg)	335 ± 36 ^a^	317 ± 22 ^a^	349 ± 27 ^a,b^	380 ± 14 ^b^	389 ± 28 ^b^
Soleus (mg)	204 ± 13	199 ± 15	198 ± 18	203 ± 10	213 ± 9
Gastrocnemius (g)	1.71 ± 0.16 ^a^	1.47 ± 0.10 ^b^	1.67 ± 0.18 ^a,b^	1.80 ± 0.07 ^a^	1.79 ± 0.11 ^a^
Visceral adipose tissue (g)	15.7 ± 3.3 ^a^	1.8 ± 0.5 ^b^	11.1 ± 1.4 ^a^	10.0 ± 4.4 ^a^	11.3 ± 0.9 ^a^
Subcutaneous adipose tissue (g)	18.8 ± 7.7 ^a^	3.1 ± 0.8 ^b^	7.6 ± 1.2 ^b,c^	7.3 ± 1.2 ^b,c^	8.3 ± 0.9 ^c^
Liver (g)	14.4 ± 1.4 ^a^	8.9 ± 0.4 ^b^	14.0 ± 0.6 ^a^	12.6 ± 0.5 ^a^	12.8 ± 0.4 ^a^
Heart (g)	1.77 ± 0.9 ^a^	1.51 ± 0.05 ^b^	1.88 ± 0.04 ^a^	1.82 ± 0.07 ^a^	1.86 ± 0.06 ^a^
Kidney (g)	1.70 ± 0.15 ^a^	1.42 ± 0.05 ^b^	1.79 ± 0.08 ^a^	1.69 ± 0.09 ^a^	1.76 ± 0.08 ^a^

Values are the means ± SEM. a ≠ b ≠ c at *p* < 0.05. Control rats were fed the standard diet *ad libitum* throughout the experiment. Food-restricted rats were allowed to consume 50% (12 g/day) of their spontaneous intakes with a standard diet for 12 weeks. At the end of restriction, all of the animals were refed for three weeks with either the standard diet or a diet supplemented with 5 g/100 g (5%) or 10 g/100 g (10%) of fresh bee pollen.

### 3.2. Biochemical and Cytokine Plasma Profiles

Plasma leptin concentration was significantly lower (−78%), whereas plasma adiponectin and resistin concentrations were significantly higher (+78% and +56%, respectively) in food-restricted old rats compared to controls (*p* < 0.05; Table 4). These changes were fully restored by the refeeding diets, except for leptin, which was increased, but not significantly, compared to food-restricted rats. Surprisingly, blood concentrations of some inflammatory markers, such as α2-macroglobulin, sTNF-R1 and sTNF-R2, were reduced during the food restriction period, while others, e.g., orosomucoid, were increased compared to controls (*p* < 0.05, Table 4). All such modifications returned to their baseline values, *i.e.*, not different compared to the control old rats, after refeeding irrespective of the marker considered (*p <* 0.05 *versus* the restricted old rats), with the exception of α2-macroglobulin, which was only restored with the 10% bee pollen diet (Table 4). Plasma insulin, TNFα, IL1β and IL6 showed no changes (Table 4).

### 3.3. Muscle Mitochondrial Activity

CS activity in the plantaris muscle decreased by 30% in the food-restricted old rats (*p <* 0.05 *versus* control old rats, Table 5). Refeeding period was not associated with modification of mitochondrial CS activity when using a standard diet, whereas CS activity was increased in rats refed with 5% or 10%-fresh pollen diets. The same trend was observed for HAD activity, but without reaching statistical significance. There was a general decrease (−12% to −24%) in complex II to IV activities in malnourished rats, but only complex IV reached statistical significance (*p <* 0.05 *versus* control old rats, Table 5). This decrease was only improved in refed rats receiving the pollen diets, *i.e.*, malnourished old rats showed significantly increased complex IV activity after a three-week refeed with a 10% fresh bee pollen diet in (*p <* 0.05 *versus* restricted old rats, Table 5).

**Table 4:** Plasma biochemistry and inflammatory profile after dietary restriction and refeeding with fresh bee pollen-containing diets in old rats.

	Controls	Restricted	Refed Standard	Refed 5% Pollen	Refed 10% Pollen
Insulin (ng/mL)	2.93 ± 1.22	1.36 ± 0.61	1.70 ± 0.41	1.66 ± 0.25	1.45 ± 0.13
Leptin (pg/mL)	2481 ± 555 ^a^	551 ± 89 ^b^	1061 ± 212 ^b^	1207 ± 201 ^b^	1087 ± 123 ^b^
Adiponectin (µg/mL)	12.9 ± 1.2 ^a^	23.0 ± 1.4 ^b^	15.0 ± 1.5 ^a^	14.3 ± 2.4 ^a^	15.7 ± 0.9 ^a^
Resistin (ng/mL)	23.6 ± 4.1 ^a^	36.7 ± 3.9 ^b^	27.2 ± 1.3 ^a^	24.4 ± 1.9 ^a^	22.0 ± 2.1 ^a^
Alpha2-macroglobuline (µg/mL)	14.0 ± 4.2 ^a^	10.1 ± 1.1 ^b^	11.8 ± 3.0 ^b^	11.2 ± 1.5 ^b^	14.8 ± 0.5 ^a^
Orosomucoid (µg/mL)	56 ± 7 ^a^	108 ± 14 ^b^	77 ± 8 ^a^	73 ± 6 ^a^	71 ± 9 ^a^
TNFα (pg/mL)	13.9 ± 0.5	12.9 ± 0.4	13.3 ± 0.3	12.9 ± 0.6	13.9 ± 0.4
sTNF-R1 (pg/mL)	128 ± 7 ^a^	94 ± 4 ^b^	122 ± 7 ^a^	129 ± 5 ^a^	130 ± 6 ^a^
sTNF-R2 (pg/mL)	1145 ± 75 ^a^	814 ± 30 ^b^	1212 ± 143 ^a^	1088 ± 51 ^a^	1105 ± 82 ^a^
IL1β (pg/mL)	38.4 ± 4.4	43.6 ± 13.1	37.1 ± 3.2	38.9 ± 13.8	31.4 ± 2.7
IL6 (pg/mL)	40.5 ± 7.7	32.6 ± 7.1	31.7 ± 3.2	44.6 ± 3.0	39.4 ± 6.3

Values are the means ± SEM. a ≠ b at *p <* 0.05. TNFα, tumor necrosis factor-α; sTNF-R1, soluble TNFα receptor 1; sTNF-R2, soluble TNFα receptor 2; IL1β, interleukin 1-β; IL6, interleukin 6. Control rats were fed the standard diet *ad libitum* throughout the experiment. Food-restricted rats were allowed to consume 50% (12 g/day) of their spontaneous intakes with a standard diet for 12 weeks. At the end of restriction, all of the animals were refed for three weeks with either the standard diet or a diet supplemented with 5 g/100 g (5%) or 10 g/100 g (10%) of fresh bee pollen.

**Table 5:** Muscle mitochondrial activity after dietary restriction and refeeding with pollen-containing diets in old rats.

	Controls	Restricted	Refed Standard	Refed 5% Pollen	Refed 10% Pollen
CS (µmol/min/mg proteins)	0.346 ± 0.029 ^a^	0.243 ± 0.028 ^b^	0.228 ± 0.032 ^b^	0.294 ± 0.031 ^a,b^	0.256 ± 0.034 ^a,b^
HAD (nmol/min/mg proteins)	8.45 ± 0.56	7.67 ± 0.92	7.56 ± 0.91	8.32 ± 1.44	8.73 ± 0.95
Complex II (nmol/min/mg proteins)	21.0 ± 1.3 ^a^	17.5 ± 0.8 ^a,b^	17.1 ± 1.3 ^b^	17.7 ± 2.4 ^a,b^	18.6 ± 1.5 ^a,b^
Complex III (nmol/min/mg proteins)	2.99 ± 0.64	2.61 ± 0.63	2.52 ± 0.39	1.98 ± 0.59	2.02 ± 0.45
Complex IV (nmol/min/mg proteins)	16.8 ± 1.5 ^a^	12.7 ± 1.7 ^b^	12.9 ± 1.4 ^b^	12.8 ± 0.9 ^b^	16.0 ± 2.3 ^a^

Values are the means ± SEM. a ≠ b at *p <* 0.05. CS, citrate synthase; HAD, 3-hydroxyacyl-CoA dehydrogenase. Control rats were fed the standard diet *ad libitum* throughout the experiment. Food-restricted rats were allowed to consume 50% (12 g/day) of their spontaneous intakes with a standard diet for 12 weeks. At the end of restriction, all of the animals were refed for three weeks with either the standard diet or a diet supplemented with 5 g/100 g (5%) or 10 g/100 g (10%) of fresh bee pollen.

### 3.4. Muscle Protein Synthesis Rate and Regulation

**Figure 1:**
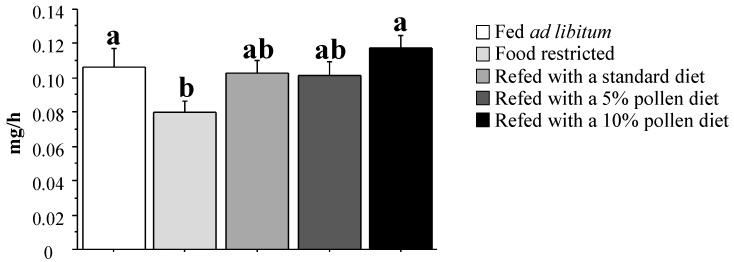
Muscle absolute synthesis rate after dietary restriction and refeeding with fresh pollen-containing diets in old rats.

**Figure 2:**
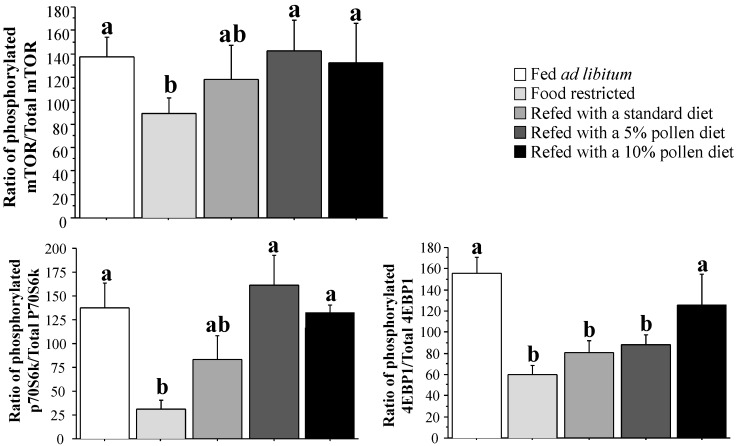
Regulation of the mTOR/p70S6k/4EBP1 signaling pathway after dietary restriction and refeeding with fresh pollen-containing diets in old rats.

A remarkable finding was that a 12-week dietary restriction led to a 26% decrease in muscle protein synthesis rate in old rats (*p <* 0.05 *versus* old control rats; Figure 1). Although the standard and 5% fresh bee pollen diets had no significant effect, the 10% fresh bee pollen diet was able to restore muscle protein synthesis during refeeding (*p <* 0.05 *versus* old restricted rats). Changes in muscle protein synthesis rate were associated with an equivalent downregulation of the mTOR/p70S6k/4EBP1 signaling pathway in restricted old rats (Figure 2). Hence, diet-restricted old rats exhibited the lowest mTOR activation rate of all animals (*p <* 0.05 *versus* control old rats; Figure 2). Of note, the mTOR phosphorylation state was better restored by refeeding with fresh bee pollen-enriched diets. In addition, although p70S6k activation and 4EBP1 activation were blunted by dietary restriction, only the 5% and 10% fresh bee pollen-enriched diets were able to restore these two controllers of protein translation (*p <* 0.05 *versus* old restricted rats; Figure 2).

Values are the means ± SEM. a ≠ b at *p <* 0.05. Control rats were fed the standard diet *ad libitum* throughout the experiment. Food-restricted rats were allowed to consume 50% (12 g/day) of their spontaneous intakes with a standard diet for 12 weeks. At the end of the restriction, all of the animals were refed for three weeks with either the standard diet or a diet supplemented with 5 g/100 g (5%) or 10 g/100 g (10%) of fresh bee pollen.

Values are the means ± SEM. a ≠ b with *p <* 0.05. Control rats were fed the standard diet *ad libitum* throughout the experiment. Food-restricted rats were allowed to consume 50% (12 g/day) of their spontaneous intakes with a standard diet for 12 weeks. At the end of the restriction, all of the animals were refed for three weeks with either the standard diet or a diet supplemented with 5 g/100 g (5%) or 10 g/100 g (10%) of fresh bee pollen.

## 4. Discussion

The aim of this study was to evaluate the biological activities of fresh monofloral bee pollen formula in improving refeeding strategies in malnourished older individuals. Experimentally, during malnutrition, early nutritional therapy can prevent or restore the depletion of essential nutrients to the body and decrease the risks of infection and other problems likely to delay recovery. In these situations, nutrient density, *i.e.*, the ability of a nutritional support to provide a complex and concentrated profile of nutrients, is vital. Since time immemorial, fresh bee pollen has been considered a good source of nourishing substances and energy. Bee pollen is normally well tolerated, but the accidental presence of allergenic pollens and substances cannot be excluded. People who are susceptible to allergies should avoid consuming pollen supplement. This product has been shown to modulate several important biological activities, making it valuable as a candidate substance to improve the efficiency of refeeding in older people.

Many elderly patients are at increased risk for malnutrition compared to other adults. It is estimated that between 2% and 16% of community-dwelling elderly are nutritionally deficient in protein and calories [27,28]. Consequently, unintended weight loss—the involuntary decline in body weight over time—is an important health risk factor among elderly persons and a strong predictor of functional limitations, admission to nursing care facilities and mortality [29,30,31]. Studies of hospitalized older patients suggest that 20%–65% of these patients suffer from nutritional deficiencies [32,33,34], and estimates put the prevalence of malnutrition in long-term care facilities at between 30% and 60% [35,36]. Experimental models using old rats to study the effects of malnutrition have already been published [14,17,18]. As is already shown [14], the model used here, *i.e.*, a 50% reduction in food intake for 12 weeks, produced similar changes to those observed during malnutrition in older humans [19,20,37], *i.e.*, a very significant decrease in body weight associated with a reduction in both lean body mass and fat mass. This was confirmed by a reduction in skeletal muscle, especially type 2 muscle, and adipose tissue weights. Of note, the decreased fat pad weight was associated with related changes in blood adipocytokines, as previously described [38,39,40]. The effect of age on body composition was evaluated in malnourished older patients [37], and in those over 70, weight loss led to a homogeneous decrease in fat-free mass and fat mass. In this situation, muscle is one of the prime targets of malnutrition [41]. The mechanisms driving this muscle depletion in malnourished older subjects are not fully understood, but are known to involve anomalies in tissue protein and energy metabolism. Accordingly, here, we observed reduced muscle protein synthesis in malnourished old rats together with a decreased ability to activate the protein translation rate, *i.e.*, decreased activation of the mTOR/p70S6k/4EBP1 signaling pathway. It has been proposed that muscle protein synthesis may be impaired by high concentrations of cytokines during malnutrition [42]. A previous study found higher concentrations of TNFα in blood and muscles of frail older subjects compared to younger ones [43]. Note that here, we found reduced blood levels of the main inflammatory factors in old malnourished rats. Most studies in malnourished older subjects have been realized from situations originating from a combination of reduced food intake and hypermetabolism, *i.e.*, increased inflammatory status. The present study is based on a model of food restriction—a dysimmunity setting that can explain the decrease in blood inflammation markers [13,15,44]. Taken together, these results show that the reduction in muscle protein synthesis during malnutrition in the elderly may be explained by more than just an exaggerated inflammatory response. Furthermore, the activity of key mitochondrial enzymes was blunted in old malnourished rats, which indicates that the deprivation of energy intake initiated in late-middle-age altered primary mitochondrial function that, in turn, depressed protein turnover in the skeletal muscle. The two phenomena are certainly interdependent, and as muscle protein turnover depends on cell energy status, it is tempting to establish a direct link between decreased mitochondrial function, *i.e.*, ATP availability in muscle cells, and a reduced protein synthesis rate. The reduced energy intake may have slowed electron flow through the electron transport chain. This hypothesis is supported by the reduced CS activity following food restriction in old rats. CS is the starting point of the Krebs cycle, and it defines the supply of electrons from reduced substrates to the mitochondrial respiratory chain. Therefore, the decrease in CS activity may lead to the observed decrease in activity of the electron transport chain complexes. However, we recently observed that a life-long, but less-severe (*i.e.*, −40%), food restriction enhanced skeletal muscle mitochondrial oxidative capacity by increasing the intrinsic function and efficiency of the mitochondrial machinery [45].

One of the most interesting findings of this study was that malnutrition-induced alterations in muscle protein and energy metabolism were reversible in old rats receiving renutrition via a fresh bee pollen-enriched diet. The well-known resistance to renutrition with age [14,15,19,20] means that supplements may be required to act as a starter of renutrition in older subjects. In addition, older persons reportedly have greater requirements of some key nutrients, such as essential amino acids, essential fatty acids and micronutrients, than adult subjects, particularly in malnutrition settings [46,47,48]. Bee pollen is considered a health food with a wide range of therapeutic properties. Here, we observed that although a short-term refeeding period was able to restore body weight and composition regardless of diet composition, a 5% or, better still, a 10% fresh bee pollen-supplemented diet showed greater efficacy to improve muscle metabolism in old malnourished rats than a standard diet. As previously [14], we showed that refeeding food-restricted rats induced a body weight recovery. The difference in weight between malnourished and refed rats roughly corresponds to the same gains in fat and fat-free mass between refed groups. Interestingly, in contrast to standard diet, the diets supplemented with 5% or 10% fresh bee pollen were able to correct the effect of undernutrition on skeletal muscle mass. The effect was limited to mixed or type-2 muscles, *i.e.*, gastrocnemius and plantaris, while type-1 soleus muscle was unaffected by either undernutrition or refeeding. Fresh bee pollen contains substantial nutrients that are the factors of anabolic properties. For instance, bee pollens are rich in essential amino acids, especially leucine, which can impart anabolic properties [49]. Recently, we clearly demonstrated that leucine supplementation was able to stimulate muscle protein synthesis in a food-deprived rat model [50]. This effect was mediated by the mTOR signaling pathway. Therefore, the positive action of the 10% fresh bee pollen-supplemented diet on muscle protein synthesis and mTOR/p70S6k/4EBP1 activation is likely one of the mechanisms of muscle restoral in old malnourished rats. In addition, fresh bee pollen contains substantial amounts of vitamins, phenolics and phytochemicals and significant quantities of other antioxidant agents [51]. Marzani *et al.* [52] assessed the effect of antioxidant supplementation on leucine-regulated protein metabolism in muscles of young and old rats and found that the ability of leucine to stimulate muscle protein synthesis was significantly decreased in old rats compared with their young counterparts. However, this defect was reversed when old rats were supplemented with antioxidants. These data revealed that antioxidant supplementation together with leucine supplementation could benefit muscle protein metabolism during aging. Thus, fresh bee pollen may have a beneficial effect on muscle protein metabolism via a synergistic effect of various nutrients, such as leucine and antioxidants. In addition, some pollen compounds, such as flavone, enhanced the gene and protein expression of Akt, a key intermediate of the insulin signaling pathway, and increased the phosphorylation state, *i.e.*, activation, of insulin receptor-β and Akt in cultured muscle cells [53]. Insulin is able to increase muscle protein synthesis [54]. Furthermore, the signaling pathway leading to the stimulation of protein synthesis in muscle cells is shared by leucine and insulin and can be stimulated by both of these mediators.

Besides an effect on the muscle protein synthesis rate, refeeding with fresh bee pollen led to increased muscle mitochondrial function in the old malnourished rats. We focused on mitochondria due to its increasingly important role in explaining aging-related loss of muscle mass and strength, in particular during malnutrition [25,55]. We observed that the 5% and 10% fresh pollen refeeding diet triggered an increase in CS activity in the plantaris muscle of food-restricted old rats. CS is a key enzyme of the Krebs cycle, and beside its functional importance for mitochondria, CS activity also is an index of mitochondrial density [26,56]. Thus, refeeding with fresh pollen-containing diets appeared to increase mitochondrial biogenesis in old malnourished rats. Accordingly, complex II and IV activities of the electron transport chain were improved, at least when using the 10% fresh pollen diet to refeed old malnourished rats. Interestingly, it was recently proposed that as complex IV activity shows all of the main regulatory mechanisms found in key metabolic enzymes, *i.e.*, isoform expression, allosteric control and phosphorylation, it likely represents the rate-limiting step in energy production [57]. Previous studies [25,58,59] have highlighted that muscle mitochondrial capacity is dependent not only on mitochondrial density, but also on the existence of qualitative differences in mitochondrial functioning. Building on our previous work [45], we propose that the fresh bee pollen-supplemented diet-induced improvement in functional mitochondria may be the result of attenuated mitochondrial oxidant emission, increased oxidant scavenging and decreased cellular oxidative damage, all of which could be expected to contribute to maintaining the functional integrity of the mitochondrial machinery. It has been reported that antioxidant potential in a lysate of erythrocytes from mice receiving a diet containing 0.1% bee pollen was elevated through increased activity of mitochondrial antioxidant enzymes [60]. The fresh bee pollen formula used here demonstrated substantial antioxidant activity, as measured through its ferric-reducing antioxidant power (FRAP) and oxygen radical absorbance capacity (ORAC). Increased antioxidant defense is consistent with the idea that bee pollen decreases oxidative damage to tissues [61], thereby preserving the function of cells and cellular components, such as mitochondria. Note that all modifications of complex activities observed in this study invariably involved complexes II and IV. Several reports have underlined the pivotal role of these particular complexes in the aging process and in the effect of antioxidant strategies [62,63,64].

## 5. Conclusions

Our study clearly shows that this fresh bee pollen formula exerts highly beneficial biological activities for the recovery of muscle protein and energy metabolism in old rats exposed to severe food restriction. We therefore conclude that fresh bee pollen could be safely included in daily diet as a supplement to enhance refeeding efficiency in human patients. The results obtained here demonstrated that fresh bee pollen possesses good anabolic and metabolic activity, suggesting that it could be useful in prevention of or recovery from malnutrition. Further clinical studies are warranted in order to elucidate the exact mechanisms underpinning these beneficial biological properties and to determine the functional significance of these findings.
